# Evaluation of Plaque Vulnerability via Combination of Hemodynamic Analysis and Simultaneous Non-Contrast Angiography and Intraplaque Hemorrhage (SNAP) Sequence for Carotid Intraplaque Hemorrhage

**DOI:** 10.3390/jpm11090856

**Published:** 2021-08-28

**Authors:** Ui Yun Lee, Hyo Sung Kwak

**Affiliations:** 1 Division of Mechanical Design Engineering, College of Engineering, Jeonbuk National University, Jeon-ju 54896, Korea; euiyun93@naver.com; 2 Department of Radiology and Research, Institute of Clinical Medicine of Jeonbuk National University, Biomedical Research Institute of Jeonbuk National University Hospital, Jeon-ju 54907, Korea

**Keywords:** carotid artery, intraplaque hemorrhage, morphological factor, hemodynamic factor, wall shear stress, computational fluid dynamics, SNAP sequence

## Abstract

The purpose of this study was to assess the vulnerability of plaque using a combination of simultaneous non-contrast angiography, intraplaque hemorrhage (SNAP) sequence, and local hemodynamic analysis in an intraplaque hemorrhage (IPH), and to evaluate the association between morphological and hemodynamic factors and IPH by comparing the IPH (presence of IPH) and non-IPH (plaque with absence of IPH) groups. In total, 27 IPH patients and 27 non-IPH patients were involved in this study, and baseline characteristics were collected. For morphological factors, diameters, and areas of the internal carotid artery (ICA), external carotid artery, and common carotid artery were measured, and bifurcation angle (α) and ICA angle (β) were also measured for comparison between the IPH group and non-IPH group. For hemodynamic factors, time-averaged wall shear stress (WSS), minimum WSS, maximum WSS, and oscillatory shear index were calculated using computational fluid dynamics (CFD) simulations. For the qualitative analysis, cross-sectional images with analyzed WSS and SNAP sequences were combined to precisely assess local hemodynamics. Bifurcation angle (α) was significantly different between the IPH and non-IPH groups (39.47 degrees vs. 47.60 degrees, *p* = 0.041). Significantly higher time-averaged WSS, minimum WSS, and maximum WSS were observed in the IPH group compared to the non-IPH group. In the IPH group, when using the combined analysis with SNAP sequences and WSS, the WSS of the region with IPH was significantly higher than the region without IPH (2.32 vs. 1.21 Pa, *p* = 0.005). A smaller bifurcation angle (α) and higher time-averaged WSS, minimum WSS, and maximum WSS were associated with IPH. The combined analysis of SNAP sequences and WSS might help to evaluate the risk of carotid IPH.

## 1. Introduction

Ischemic stroke is a major cause of disability and morbidity in adults worldwide [1]. Atherosclerosis in the carotid artery progresses via various biological mechanisms leading to the formation of multiple components. The composition of plaque is related to plaque rupture and cerebrovascular events. Intraplaque hemorrhage (IPH), a large lipid-rich necrotic core (LRNC), and a thin fibrous cap increase the risk of rupture. In contrast, the presence of calcification is associated with plaque stability [2,3,4,5].

Among the plaque components, IPH has six-fold susceptibility to the development of cerebrovascular events [6,7]. Carotid IPH is also vulnerable to embolization regardless of the severity of stenosis [8]. Recently, the simultaneous non-contrast angiography and intraplaque hemorrhage (SNAP) sequence has been employed as a useful tool to detect IPH from a small size. Early detection of carotid IPH using imaging techniques such as the SNAP sequence can reduce the risk of stroke and can suggest a timely treatment method [9]. Kim et al. [9] used the SNAP sequence as a one-step examination method to detect carotid IPH and vertebrobasilar artery IPH.

The mechanism of initiation and progression of atherosclerosis in the carotid artery is still not clearly elucidated. It is known that hemodynamic factors affect plaque vulnerability. Plaque formation or progression can be explained by flow variability and wall shear stress (WSS) in the bulb of the carotid artery [4,10]. Tuenter et al. [4] conducted a subject-specific computational fluid dynamics (CFD) study and found that IPH is associated with higher maximum wall shear stress. Huang et al. [11] also reported that higher wall shear stress is related to IPH. However, it is controversial whether low WSS or high WSS is associated with IPH [12]. Moreover, as hemodynamic factors represent the interplay between blood flow and morphological factors, it is necessary to investigate not only hemodynamic factors (i.e., WSS) but also morphological factors in carotid bifurcation [2]. 

Thus, the purpose of the present study was to evaluate plaque vulnerability by combining SNAP sequences and local hemodynamic analysis in IPH. An association between risk factors (morphological and hemodynamic factors) and IPH was identified by comparing IPH and non-IPH groups. Moreover, morphological and hemodynamic factors were quantitatively analyzed to characterize IPH.

## 2. Materials and Methods

### 2.1. Study Population

The present study was approved by the Institutional Review Board of Jeonbuk National University Hospital (The Ethics Committee of Jeonbuk National University Hospital), and informed consent was obtained from all patients prior to brain imaging (JUH 2017-10-007). From September 2018 to April 2020, patients who had neurological symptoms such as dizziness, headache, vertigo, or giddiness underwent brain magnetic resonance imaging (MRI) with SNAP sequence and MR angiography to evaluate the presence of IPH. Based on brain MRI with the SNAP sequence, the patients were divided into an IPH group (presence of IPH) and a non-IPH group (plaque with absence of IPH). There were 148 patients in the IPH group and 138 patients in the non-IPH group. Among the patients, the final group was selected based on the following exclusion criteria: (1) poor image quality to evaluate the plaque, (2) insufficient quality of raw data for CFD simulations, and (3) more than 15% stenosis according to the North American Symptomatic Carotid Endarterectomy Trial (NASCET) criteria. According to the exclusion criteria, 27 patients in the IPH group (19 males, 8 females; age range from 70–78 years) and 27 patients in the non-IPH group (19 males, 8 females; age range from 70.5–78 years) were selected for the final groups of our study. For the comparison between the two groups, similarly aged and gendered patients were obtained to minimize age and gender differences. 

Baseline characteristics of the finally selected patients were collected. Medical histories of hypertension, diabetes mellitus, dyslipidemia, history of cardiovascular disease, history of cerebrovascular disease, smoking, and atrial fibrillation were collected and compared between IPH and non-IPH groups. As shown in Table 1, there were no significant differences between the two groups. 

### 2.2. MR Imaging Acquisition

Acquisition of MR imaging was performed with a 3 T MRI scanner (Achieva; Philips Medical Systems, Amsterdam, The Netherlands) with a 16-channel head coil. Imaging acquisition protocols were as follows:

(1) MRI with SNAP sequence: repetition time/echo time (TR/TE) 10/4.7 ms, flip angle 11°, field-of-view (FOV) 149 × 149 mm, matrix = 187 × 216, acquisition time 3 min 30 s;
(2) TOF-MRA: TR/TE 21/3.6 ms, flip angle 18°, slice thickness 0.6 mm, FOV 172 × 200 mm, matrix = 384 × 198, acquisition time 4 min 25 s, echo train length 1.

### 2.3. MR Imaging Review

The review of the SNAP sequences was conducted by two neuroradiologists with more than 30 years of experience and without knowing the purpose of this study. The image quality and presence of IPH were assessed. Plaque was defined as a thickness greater than 2 mm on more than two slices. IPH was characterized as a signal intensity higher than 200% compared to the surrounding muscle. 

### 2.4. Quantification of Morphological Factors

Based on obtained TOF-MRA Digital Imaging and Communications in Medicine format data, 27 blood vessels of the IPH group and 27 blood vessels of the non-IPH group were converted from two-dimensional to three-dimensional (3D) shapes using thresholding and the 3D calculation method in Mimics software (version 21.0; Materialise NV, Leuven, Belgium). Since accurate geometry should be obtained and used for measurements of morphological factors and CFD analysis, the two neuroradiologists with more than 30 years of experience confirmed all segmentation processes. Unnecessary branches of the external carotid artery (ECA) were removed, and the converted 3D shapes were saved in stereolithography (STL) file format. 

**Figure 1:**
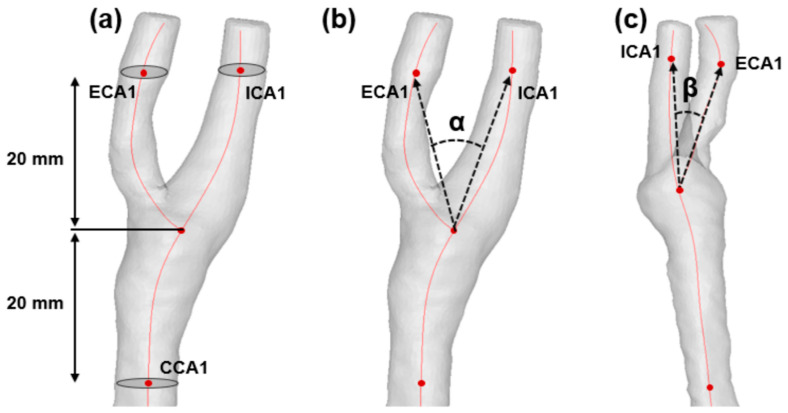
Definition of morphological factors. ICA, internal carotid artery; ECA, external carotid artery; CCA, common carotid artery. (**a**) Automatically generated centerline is shown with a red line. ICA1, ECA1, and CCA1 refer to the points 20 mm away from the carotid bifurcation. Diameters and areas were measured at the points (ICA1, ECA1, and CCA1). (**b**) Bifurcation angle (α) was measured. (**c**) ICA angle (β) was measured.

Using Mimics software, diameters and areas of the internal carotid artery (ICA), external carotid artery (ECA), and common carotid artery (CCA) were measured from a point 20 mm away from the carotid bifurcation after the centerline was generated on the carotid artery (ECA1, ICA1, and CCA1) as shown in Figure 1a. The bifurcation angle (α) and ICA angle (β) were also measured to compare between the IPH and non-IPH groups. The bifurcation angle (α) was defined as the angle between the projection of ECA and ICA vectors of the carotid artery (Figure 1b). The ICA angle (β) was characterized as the angle between the projection of the CCA and ICA vector on the branch plane (Figure 1c) [13,14].

### 2.5. Computational Fluid Dynamics

The 54 carotid bifurcation geometries in STL format were imported to the simulations of blood flow dynamics using COMSOL Multiphysics 5.2a software (COMSOL Inc., Burlington, MA, USA). The incompressible and laminar Newtonian model with constant viscosity of 0.0035 Pa∙s was utilized for the CFD simulation [8]. Blood density was assumed to be 1066 kg/m^3^. A rigid no-slip boundary condition was set for the wall condition [15,16]. For the boundary condition of CCA and ICA, the published flow rate was applied, and patient-specific areas of the CCA and ICA were used to calculate velocity [17]. A traction-free boundary condition was set for the ECA outlet. Three cardiac cycles were simulated, and the second cardiac cycle was employed to calculate hemodynamic factors to avoid numeric artifacts [18,19]. For each carotid model, CFD calculation and post-processing time took from approximately 3 to 6 h.

### 2.6. Analysis of Hemodynamic Factors

Based on the results of time-dependent CFD simulations, time-averaged WSS, minimum WSS, maximum WSS, and oscillatory shear index (OSI) were quantified for comparison between the IPH and non-IPH groups. The WSS-based hemodynamics factors were the data from the surface of the diseased area. Time-averaged WSS was defined as the averaged WSS of one cardiac cycle. For the IPH group and non-IPH group, the plaque location was different for each patient. Therefore, the volumetric time-averaged WSS was calculated from the start point to the end point of the plaque which was specified by the neuroradiologists. The minimum WSS and maximum WSS were defined as the lowest wall shear stress and the highest wall shear stress during one cardiac cycle, respectively. The OSI was defined as the results of directional changes of wall shear stress in one cardiac cycle. 

### 2.7. Combined Analysis Using SNAP Sequence and WSS

**Figure 2:**
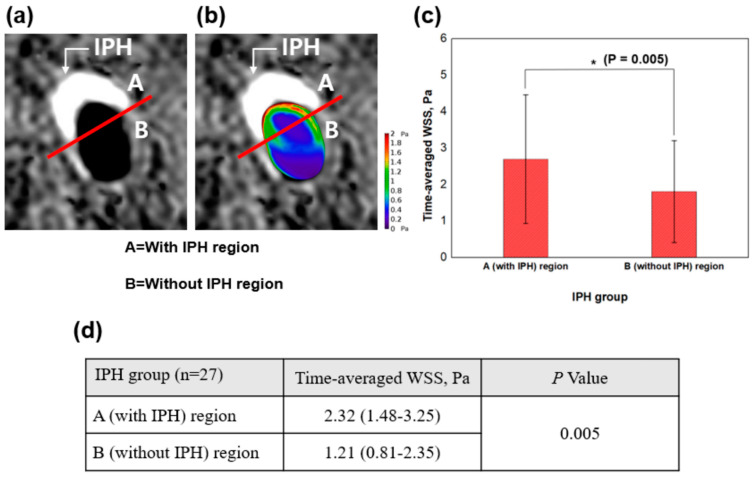
Combined analysis using SNAP sequences and wall shear stress (WSS). IPH, intraplaque hemorrhage; WSS, wall shear stress. (**a**) The SNAP sequence was divided into an A (with IPH) region and a B (without IPH) region. On the SNAP sequence, the white arrow refers to the brighter region (IPH) compared to surrounding muscles. (**b**) An example of a combination image of SNAP and WSS. (**c**) Time-averaged WSS of the A (with IPH) region and the B (without IPH) region is compared and shown with a bar graph. * *p* values indicate differences between the A (with IPH) region and the B (without IPH) region. (**d**) The quantitative comparison between the A (with IPH) region and the B (without IPH) region is shown.

In Figure 2a, the SNAP sequence of the IPH group was divided into A (with IPH) and B (without IPH) regions by drawing a straight line. The A (with IPH) region referred to the presence of IPH in the SNAP sequence, and the B (without IPH) region referred to the absence of IPH in the SNAP sequence. The time-averaged WSS for the divided regions of A and B was calculated, respectively. In Figure 2a,b, the IPH region which was brighter than the surrounding muscles marked with a white arrow.

We overlapped the SNAP sequences with cross-sectional images of WSS in the IPH group to analyze plaque vulnerability. The time-averaged WSS was quantitatively analyzed for each region (A and B regions), and the results were compared between the A (with IPH) region and B (without IPH) region. The combined analysis using the SNAP sequences and cross-sectional images of WSS focused on the following two perspectives: first, the analysis was performed to precisely visualize local WSS at the presence of the IPH region by combining SNAP sequences and cross-sectional WSS; second, the analysis was conducted to check the difference of WSS between the with-IPH region and the without-IPH region in the IPH group. The overlapped resultant image is shown in Figure 2b as an example.

### 2.8. Statistical Analysis

Data with non-normal distributions were expressed as medians (interquartile range) and were compared with the Mann–Whitney U test. Data with non-normal distributions were expressed as mean ± standard deviations and were compared between the groups using the Student’s *t*-test. Categorical variables were shown as frequencies and percentages and were compared using Pearson’s chi-square test. 

## 3. Results

### 3.1. Comparison of Morphological Factors between IPH and Non-IPH Groups

Table 2 shows the comparison of morphological factors between the IPH and non-IPH groups. Among the morphological factors, a significantly smaller bifurcation angle (α) was observed in the IPH group compared to the non-IPH group (39.47 degrees vs. 47.60 degrees, *p* = 0.041). The ICA angle (β) of the IPH group was also smaller than that of the non-IPH group, but no significant difference was found (*p* = 0.156).

### 3.2. Hemodynamic Characteristics of IPH

A comparison of quantified WSS between the IPH and non-IPH groups was conducted (Table 2). The time-averaged WSS was found to be significantly higher in the IPH group compared to the non-IPH group (1.84 Pa vs. 1.10 Pa, *p* = 0.007). The minimum WSS (0.60 Pa vs. 0.42 Pa, *p* = 0.015) and maximum WSS (4.27 Pa vs. 2.41 Pa, *p* = 0.005) were observed to be significantly higher in the IPH group compared to the non-IPH group. The OSI of the IPH group appeared to have a higher value compared to that of the non-IPH group, but no significant difference was found (0.39 vs. 0.38, *p* = 0.494).

Figure 2b shows the representative case of the combined analysis using SNAP sequences and WSS. The distribution of higher WSS was found in the A (with IPH) region compared to the B (without IPH) region. In the IPH group (*n* = 27), a statistically significant difference in time-averaged WSS was found between the A (with IPH) region and B (without IPH) region (2.32 vs. 1.21 Pa, *p* = 0.005) as shown in Figure 2c,d. 

**Figure 3:**
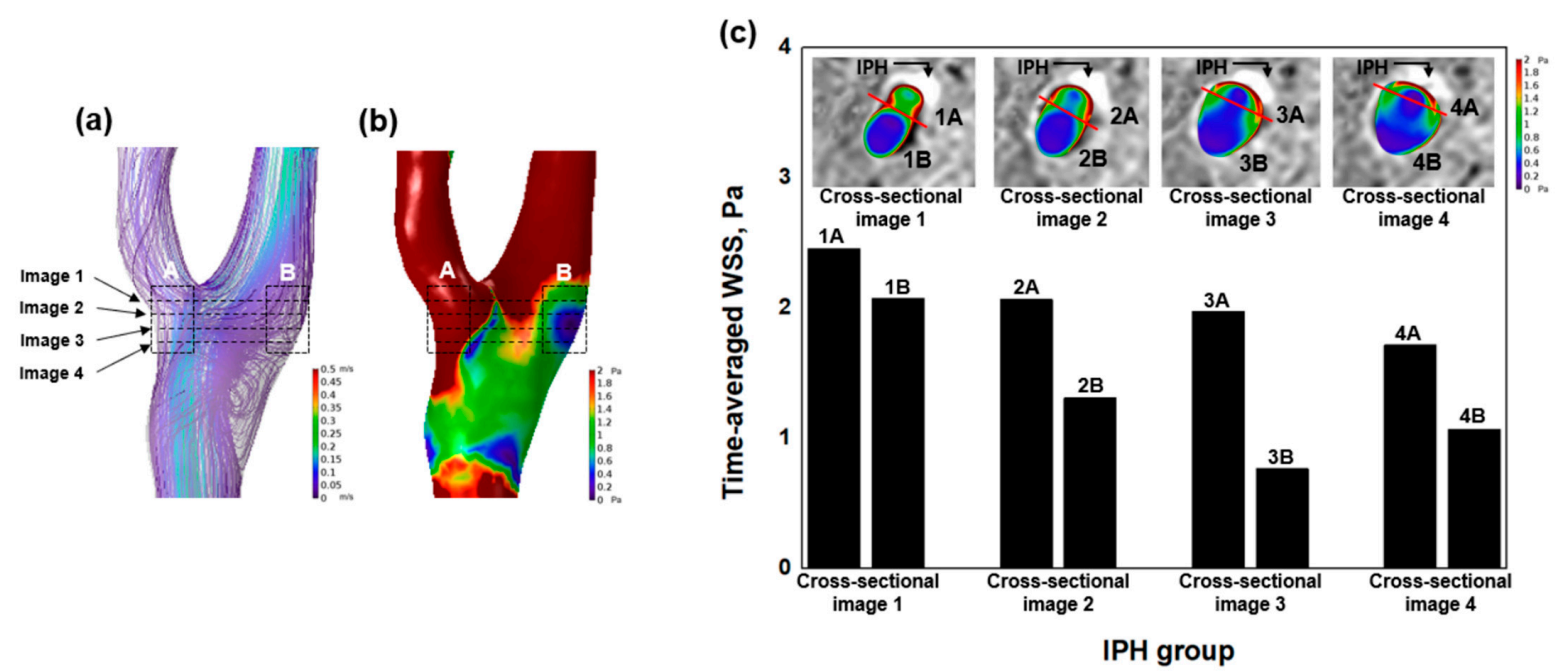
Detailed combined analysis between SNAP and local hemodynamic with a representative case. (**a**) Streamlines of the carotid artery with ranges from 0 m/s to 0.5 m/s are shown. Image 1, image 2, image 3, and image 4 are pointed to with black arrows, and the dashed black lines refer to each image. The A (with IPH) region and the B (without IPH) region are marked with a box. (**b**) Wall shear stress (WSS) of the carotid artery with ranges from 0 Pa to 2 Pa is shown. (**c**) The comparison of quantitative analysis (time-averaged WSS) between the A (with IPH) region and the B (without IPH) region is shown with a bar graph. The areas of IPH are marked with black arrows. All A (with IPH) regions had higher time-averaged WSS compared to B (without IPH) regions.

For more detailed analysis, Figure 3 shows a representative case of the IPH group. Less flow disturbance (Figure 3a) and higher WSS (Figure 3b) were observed at the A (with IPH) region compared to the B (without IPH) region. We analyzed combined images between the SNAP sequences and WSS on each cross-sectional image (Figure 3c). From cross-sectional images 1 to 4, all A (with IPH) regions had higher WSS compared to B (without IPH) regions. For example, at cross-sectional image 1, 1A had higher WSS than 1B (2.45 Pa vs. 2.06 Pa). 

## 4. Discussion

Our study demonstrated differences in morphological and hemodynamic factors between IPH and non-IPH groups. A smaller bifurcation angle (α) and higher time-averaged, minimal, and maximal WSS were found in the IPH group compared to the non-IPH group. For the analysis of local hemodynamic characteristics of IPH, we combined SNAP sequences and WSS data, and the difference between regions with IPH and regions without IPH was statistically significant. 

### 4.1. High WSS and IPH

In our study consisting of patients with only mild or no carotid artery stenosis, the IPH group presented higher time-averaged, minimum, and maximum WSS than the non-IPH group. The smaller bifurcation angle (α) in the IPH group may have contributed to the flow patterns within the carotid artery in which the endothelium is exposed to high WSS [2]. Yet, it is not fully understood how IPH is influenced by WSS. Eshtehardi et al. [20] suggested that high WSS transforms the compositions of plaque into a vulnerable form via angiogenesis. The resulting neovessels can be deformed by local hemodynamic forces as mentioned in a previous study reported by Lu et al. [21]. Neovessels deformed by high WSS may possess higher susceptibility to leakage, allowing hemorrhage inside plaques [8].

### 4.2. Combination of Hemodynamic Analysis and SNAP Sequence

Wang et al. [22] conducted animal experiments using positive and negative contrast agents to map ischemic regions in MRI. Moerman et al. [23] performed a combined analysis with histology sections and WSS to find the correlation between WSS and plaque thickness. Similar to Moerman et al.’s combined analysis, we conducted the combined analysis of SNAP sequences and WSS data. SNAP sequences have expanded our diagnostic ability to detect small-sized IPH but currently, they have limitations in predicting prognosis. Novel tools displaying hemodynamic data may have an additional prognostic value. Assessment for the risk of stroke using the SNAP sequence may be advanced by calculation and visualization of local WSS on images obtained from the SNAP sequence. WSS and IPH, localized and evaluated together at an early stage of the atherosclerotic process, may provide clinical benefit in predicting and minimizing the risk of stroke through an adjustment of the therapeutic strategy. 

In this study, errors could occur during MR imaging acquisition, MR imaging review, segmentation, cross-sectional cuts, and combining SNAP sequences and local hemodynamic data. In order to minimize the errors and confirm the reproducibility, two experienced neuroradiologists (with more than 30 years experience) were involved throughout the entire process of our study. These processes were repeated twice to check the reproducibility and to confirm the similar results.

### 4.3. Limitations of the Study

First, since the number of patients was small in this study, several limitations occurred as follows: (1) the sample size of our study decreased due to the exclusion criteria, which resulted in its limited statistical power, and (2) there was a limitation to suggest cut off values for WSS predicting intraplaque hemorrhage prone to rupture. Second, although a patient-specific SNAP sequence was used, the inflow condition for the CFD analysis was not patient-specific because we applied the inflow condition based on the reference paper (older adults with little or no disease in carotid artery). Third, due to the insufficient patient data, we set the wall conditions as rigid for the CFD simulation. However, since plaque is usually known to be thick, the CFD simulations should not have been significantly affected [4]. Lastly, although blood flow behaves as a non-Newtonian fluid, we assumed blood properties as a Newtonian fluid due to the lack of measured blood viscosity data. 

## 5. Conclusions

Our study investigated the influence of hemodynamic and morphological factors on co-existing conditions on IPH. Compared to the non-IPH group, the IPH group presented higher wall shear stress and a smaller bifurcation angle (α). Simultaneous visualization of WSS and SNAP sequences might help to predict prognosis and assess the risk of IPH for a carotid artery with mild or no stenosis. 

## Figures and Tables

**Table 1:** Baseline Characteristics of Patients.

Variables	IPH Group (*n* = 27)	Non-IPH Group (*n* = 27)	*p* Value
Age, median (IQR)	72.00 (70.00–78.00)	72.00 (70.50–78.00)	0.952
Male, *n* (%)	19 (70.37)	19 (70.37)	1.000
Medical history, *n* (%)			
Hypertension	19 (70.37)	22 (81.48)	0.524
Diabetes mellitus	11 (40.74)	14 (51.85)	0.585
Dyslipidemia	9 (33.33)	4 (14.81)	0.203
History of cardiovascular disease	3 (11.11)	6 (22.22)	0.465
History of cerebrovascular disease	9 (33.33)	5 (18.52)	0.352
Smoking	6 (22.22)	4 (14.81)	0.726
Atrial fibrillation	2 (7.41)	2 (7.41)	1.000

Values in the table are presented as median (IQR) or *n* (%). The data with a non-normal distribution are shown with median (IQR). *p* values indicate differences between IPH and non-IPH groups. IQR, interquartile range; IPH, intraplaque hemorrhage.

**Table 2:** Comparison of Morphological and Hemodynamic Risk Factors.

Morphological Variables	IPH Group (*n* = 27)	Non-IPH Group (*n* = 27)	*p* Value
ICA diameter, mm	4.80 (4.38–5.05)	5.04 (4.64–5.86)	0.115
ECA diameter, mm	3.91 ± 0.72	4.18 ± 0.73	0.174
CCA diameter, mm	6.73 ± 0.97	7.12 ± 0.96	0.148
ICA area, mm^2^	17.91 (14.96–19.93)	19.75 (16.49–26.63)	0.132
ECA area, mm^2^	12.25 ± 4.52	13.96 ± 4.95	0.189
CCA area, mm^2^	36.16 ± 10.61	40.51 ± 10.95	0.145
Bifurcation angle (α), degree°	39.47 (29.27–48.97)	47.60 (38.94–53.77)	0.041
ICA angle (β), degree°	44.64 (32.05–54.64)	48.85 (40.19–56.91)	0.156
Hemodynamic variables			
Time-averaged WSS, Pa	1.84 (1.21–2.77)	1.10 (0.80–1.58)	0.007
Minimum WSS, Pa	0.60 (0.35–1.05)	0.42 (0.27–0.50)	0.015
Maximum WSS, Pa	4.27 (3.11–5.76)	2.41 (2.07–4.04)	0.005
Oscillatory shear index	0.39 (0.34–0.41)	0.38 (0.35–0.40)	0.494

Values in the table are presented as mean ± standard deviation (for data with normal distribution) or median, interquartile range (for data with non-normal distribution). *p* values indicate differences between the IPH and non-IPH groups. IPH, intraplaque hemorrhage; ICA, internal carotid artery; ECA, external carotid artery; CCA, common carotid artery; WSS, wall shear stress.

## Data Availability

The data presented in this study are available upon request from the corresponding author. The data are not publicly available due to privacy restrictions.

## References

[1] Noh S.-M., Kang H.G. (2019). Clinical significance of the internal carotid artery angle in ischemic stroke. Sci. Rep..

[2] Jiang P., Chen Z., Hippe D.S., Watase H., Sun B., Lin R., Yang Z., Xue Y., Zhao X., Yuan C. (2020). Associations between Carotid Bifurcation Geometry and Atherosclerotic Plaque Vulnerability: A Chinese Atherosclerosis Risk Evaluation II Study. Mol. Cell. Biomech..

[3] Selwaness M., Bouwhuijsen Q.V.D., van Onkelen R.S., Hofman A., Franco O.H., van der Lugt A., Wentzel J.J., Vernooij M. (2014). Atherosclerotic Plaque in the Left Carotid Artery Is More Vulnerable than in the Right. Stroke.

[4] Tuenter A., Selwaness M., Lorza A.A., Schuurbiers J., Speelman L., Cibis M., van der Lugt A., de Bruijne M., van der Steen A., Franco O. (2016). High shear stress relates to intraplaque haemorrhage in asymptomatic carotid plaques. Atherosclerosis.

[5] Groen H.C., Gijsen F.J., van der Lugt A., Ferguson M.S., Hatsukami T.S., van der Steen A.F., Yuan C., Wentzel J.J. (2007). Plaque rupture in the carotid artery is localized at the high shear stress region: A case report. Stroke.

[6] Saam T., Hetterich H., Hoffmann V.S., Yuan C., Dichgans M., Poppert H., Koeppel T., Hoffmann U., Reiser M.F., Bamberg F. (2013). Meta-Analysis and Systematic Review of the Predictive Value of Carotid Plaque Hemorrhage on Cerebrovascular Events by Magnetic Resonance Imaging. J. Am. Coll. Cardiol..

[7] Kwak H.S., Yang H.J., Hwang S.B., Chung G.H. (2017). Carotid Wall Imaging with Routine Brain MRI to Facilitate Early Detection of Carotid Plaque and Intraplaque Hemorrhage. J. Stroke.

[8] Dai Y., Qian Y., Zhang M., Li Y., Lv P., Tang X., Javadzadegan A., Lin J. (2019). Associations between local haemodynamics and carotid intraplaque haemorrhage with different stenosis severities: A preliminary study based on MRI and CFD. J. Clin. Neurosci..

[9] Kim M.J., Kwak H.S., Hwang S.B., Chung G.H. (2021). One-step evaluation of intraplaque hemorrhage in the carotid artery and vertebrobasilar artery using simultaneous non-contrast angiography and intraplaque hemorrhage. Eur. J. Radiol..

[10] Kamenskiy A.V., MacTaggart J.N., Pipinos I.I., Bikhchandani J., Dzenis Y.A. (2012). Three-Dimensional Geometry of the Human Carotid Artery. J. Biomech. Eng..

[11] Huang X., Teng Z., Canton G., Ferguson M., Yuan C., Tang D. (2010). Intraplaque hemorrhage is associated with higher structural stresses in human atherosclerotic plaques: An In Vivo MRI-based 3d fluid-structure interaction study. Biomed. Eng. Online.

[12] Eshtehardi P., Teng Z. (2016). Protective or destructive: High wall shear stress and atherosclerosis. Atherosclerosis.

[13] Thomas J.B., Antiga L., Che S.L., Milner J.S., Hangan Steinman D.A., Spence J.D., Rutt B.K., Steinman D.A. (2005). Variation in the carotid bifurcation geometry of young versus older adults: Implications for geometric risk of atherosclerosis. Stroke.

[14] Jeon S.J., Kwak H.S., Chung G.H. (2018). Widening and Rotation of Carotid Artery with Age: Geometric Approach. J. Stroke Cerebrovasc. Dis..

[15] Ngo M.T., Kim C.I., Jung J., Chung G.H., Lee D.H., Kwak H.S. (2019). Four-Dimensional Flow Magnetic Resonance Imaging for Assessment of Velocity Magnitudes and Flow Patterns in The Human Carotid Artery Bifurcation: Comparison with Computational Fluid Dynamics. Diagnostics.

[16] Dai Y., Lv P., Javadzadegan A., Tang X., Qian Y., Lin J. (2018). Hemodynamic analysis of carotid artery after endarterectomy: A preliminary and quantitative imaging study based on computational fluid dynamics and magnetic resonance angiography. Quant. Imaging Med. Surg..

[17] Hoi Y., Wasserman B.A., Xie Y.J., Najjar S.S., Ferruci L., Lakatta E., Gerstenblith G., A Steinman D. (2010). Characterization of volumetric flow rate waveforms at the carotid bifurcations of older adults. Physiol. Meas..

[18] Lee S.-W., Antiga L., Spence J.D., Steinman D.A. (2008). Geometry of the Carotid Bifurcation Predicts Its Exposure to Disturbed Flow. Stroke.

[19] Compagne K., Dilba K., Postema E., Van Es A., Emmer B., Majoie C., Van Zwam W., Dippel D., Wentzel J., Van Der Lugt A. (2019). Flow Patterns in Carotid Webs: A Patient-Based Computational Fluid Dynamics Study. Am. J. Neuroradiol..

[20] Eshtehardi P., Brown A.J., Bhargava A., Costopoulos C., Hung O.Y., Corban M.T., Hosseini H., Gogas B.D., Giddens D.P., Samady H. (2017). High wall shear stress and high-risk plaque: An emerging concept. Int. J. Cardiovasc. Imaging.

[21] Lu J., Duan W., Qiao A. (2015). Finite element analysis of mechanics of neovessels with intraplaque hemorrhage in carotid atherosclerosis. Biomed. Eng. Online.

[22] Zong X., Wang P., Kim S.-G., Jin T. (2014). Sensitivity and Source of Amine-Proton Exchange and Amide-Proton Transfer Magnetic Resonance Imaging in Cerebral Ischemia. Magn. Reson. Med..

[23] Moerman A.M., Dilba K., Korteland S., Poot D.H.J., Klein S., Van Der Lugt A., Rouwet E.V., Van Gaalen K., Wentzel J.J., Van Der Steen A.F.W. (2019). An MRI-based method to register patient-specific wall shear stress data to histology. PLoS ONE.

